# A Systematic Review of Nanomedicine in Glioblastoma Treatment: Clinical Efficacy, Safety, and Future Directions

**DOI:** 10.3390/brainsci13121727

**Published:** 2023-12-18

**Authors:** Minaam Farooq, Gianluca Scalia, Giuseppe E. Umana, Urja A. Parekh, Faiza Naeem, Sayeda Fatima Abid, Muhammad Hammad Khan, Shah Gul Zahra, Hrishikesh P. Sarkar, Bipin Chaurasia

**Affiliations:** 1 Department of Neurological Surgery, Weill Cornell Medicine, New York Presbyterian Hospital, New York, NY 10021, USA; minaamkemu2018@gmail.com; 2 Neurosurgery Unit, Department of Head and Neck Surgery, Garibaldi Hospital, 95123 Catania, Italy; 3 Department of Neurosurgery, Gamma Knife and Trauma Center, Cannizzaro Hospital, 95126 Catania, Italy; umana.nch@gmail.com; 4 German Cancer Research Center, 69120 Heidelberg, Germany; urjaparekh@gmail.com; 5 Department of Neurosurgery, King Edward Medical University, Lahore 54000, Pakistan; faizanaeem312@gmail.com (F.N.); fatimasyed@kemu.edu.pk (S.F.A.); hammadkhan@kemu.edu.pk (M.H.K.); shahgulzahra4@gmail.com (S.G.Z.); 6 Department of Neurological Sciences, Kokilaben Dhirubhai Ambani Hospital, Mumbai 400053, India; hrishikesh.sarkar@hotmail.com; 7 Department of Neurosurgery, Neurosurgery Clinic, Birgunj 44300, Nepal; trozexa@gmail.com

**Keywords:** glioblastoma, nanomedicine, nanoparticles, quality of life, theranostics, molecular markers

## Abstract

(1) Background: Glioblastoma (GBM) is categorized as a grade IV astrocytoma by the World Health Organization (WHO), representing the most aggressive and prevalent form of glioma. It presents a significant clinical challenge, with limited treatment options and poor prognosis. This systematic review evaluates the efficacy and safety of various nanotherapy approaches for GBM and explores future directions in tumor management. Nanomedicine, which involves nanoparticles in the 1–100 nm range, shows promise in improving drug delivery and targeting tumor cells. (2) Methods: Following PRISMA guidelines, a systematic search of databases including Google Scholar, NCBI PubMed, Cochrane Library, and ClinicalTrials.gov was conducted to identify clinical trials on GBM and nanomedicine. The primary outcome measures were median overall survival, progression-free survival, and quality of life assessed through Karnofsky performance scores. The safety profile was assessed by adverse events. (3) Results: The analysis included 225 GBM patients, divided into primary and recurrent sub-populations. Primary GBM patients had a median overall survival of 6.75 months, while recurrent GBM patients had a median overall survival of 9.7 months. The mean PFS period was 2.3 months and 3.92 months in primary GBM and recurrent GBM patients, respectively. Nanotherapy showed an improvement in quality of life, with KPS scores increasing after treatment in recurrent GBM patients. Adverse events were observed in 14.2% of patients. Notably, Bevacizumab therapy exhibited better survival outcomes but with a higher incidence of adverse events. (4) Conclusions: Nanotherapy offers a modest increase in survival with fewer severe side effects. It shows promise in improving the quality of life, especially in recurrent GBM patients. However, it falls short in terms of overall survival compared to Bevacizumab. The heterogeneous nature of treatment protocols and reporting methods highlights the need for standardized multicenter trials to further evaluate the potential of nanomedicine in GBM management.

## 1. Introduction

Glioblastoma (GBM) is a fast-growing central nervous system (CNS) tumor arising from the supportive cells called the glial cells of the CNS, representing the most aggressive and prevalent form of glioma, as defined by the National Cancer Institute (NCI). GBM is the most aggressive of all brain tumors and is defined by the World Health Organization (WHO) as a grade 4 astrocytoma [1]. Despite the implementation of this aggressive multidisciplinary strategy, the median survival time is approximately half a year, and the 5-year survival rate is only around 10%, or potentially even lower [2,3,4]. The incidence of GBM ranges from two to three adults per 100,000 a year. It is so prevalent that it alone accounts for more than 52% of all tumors in the brain. It occurs at an old age, with a median age of occurrence at 64 years, and occurs more commonly among men [5]. Research has shown that glioblastoma (GBM) cells release extracellular vesicles (EVs) that carry cargo selectively enriched for oncogenic functions, with an estimated 10,000 EVs released from a single GBM cell over 48 h [6]. Significantly, EVs derived from glial tumor cells are taken up in both an autocrine and paracrine manner, indicating a role for these vesicles in intercellular communication within the brain. Indeed, the uptake of these oncogenic EVs by malignant cells has been linked to tumor progression [7,8], invasion [9], angiogenesis [6], treatment resistance, and the promotion of tumor progression through the modulation of the microenvironment when taken up by surrounding normal cells [6,10,11,12]. Standard treatment protocols for newly diagnosed glioblastoma (GBM) generally involve maximal safe surgical resection [13], followed by adjuvant therapy such as radiotherapy and chemotherapy (external beam radiotherapy combined with temozolomide) [2,3,14], or the utilization of tumor-treating fields (TTFields) [4,15], along with their appropriate combined regimens. Despite several advancements in the field of newer drug delivery systems (DDSs), such as hyaluronic acid-based drug nanocarrier drug delivery systems, red blood cell membrane-camouflaged nanoparticle drug delivery systems, and hexagonal boron nitride nanosheet drug delivery systems, there is still a significant need for more effective therapeutic strategies to improve the prognosis and survival outcomes of patients with glioblastoma [16].

Nanomedicine refers to the use of minutely sized molecules, often biomolecules, ranging in size from 1 to 100 nm. These nanoparticles are becoming increasingly used for various purposes in therapeutic and diagnostic medicine. There is a growing literature on the synergistic use of nanoparticles and bulk chemotherapeutics for cancer treatment [17]. Currently, there are over 50 nanomedicines approved for use by the Food and Drug Authority (FDA) [18]. Several magnetic, optical, electrical, and mechanical properties make them suitable for use in certain situations, in preference to their bulk counterparts [19]. Nanoparticles (NPs) are used in surgery, targeted drug delivery, genetic engineering, biosensing and detection of biomarkers, artificial implants, diagnostics and screening, and tissue engineering, among many other uses [20]. Nanomedicine exhibits better cell specificity and sensitivity in diagnostics and better cell-specific toxicity against cancerous lesions and is incorporated into several treatment strategies. These advantages have allowed for the emergence of precision medicine and “theranostics”, the combination of therapeutics and diagnostics [21,22]. An interesting advancement is the use of organic nanoparticles that are purposely made for their ability to cross the blood–brain barrier (BBB). These include micelles, liposomes, and protein- and lipid-based nanoparticles [23,24,25]. Previously existing reviews of NPs used for the treatment of GBM consider and weigh the efficacy and safety of one form of nanotherapy [26,27]. While this approach has its merits in exploring a single form of therapy in depth, it lacks a comparison of these studies to current novel approaches. The aim of our systematic review is thus to consider the newer technologies available currently for GBM therapy at large, and to consider the effectiveness and risks of one therapy over the others, to add to the existing literature with the most up-to-date findings. Furthermore, it aims to investigate future directions in tumor management by highlighting the potential trajectory that the field of nanomedicine and theranostics may follow in tumor management.

## 2. Materials and Methods

The PRISMA guidelines were followed to perform a systematic review [28]. PRISMA (PROSPERO) registration was also performed for the systematic review (Registration ID: CRD42023479690). A thorough search for articles describing clinical trials employing nanotechnology to treat GBM was made on Google Scholar, NCBI PubMed, Cochrane Library, and ClinicalTrials.gov. Mesh terms used were as follows: “Glioblastoma multiforme” AND “Nanotechnology” OR “Theranostics” OR “Nanoparticles” OR “Liposomes” AND “Clinical Trials”. Only those studies published in English from 2001 to 2020 were included. Ongoing clinical trials and those with a follow-up period of less than 8 months were excluded. Our PICO (population, intervention, comparator, and outcome) framework only included patients older than 18 years who had primary and/or recurrent glioblastoma (population) and were treated with nanoparticles coated with anti-cancer drugs (intervention), compared against conventional therapies such as Bevacizumab (comparator). The primary outcome measure was median overall survival, and secondary outcome measures were the progression-free survival rate (PFS-12), progression-free survival (PFS), and quality of life assessed via Karnofsky performance scores (KPS).

Patients suffering from gliomas other than GBM were excluded from the study as were those patients who were younger than 18 years of age. The safety profile was assessed by evaluating the side effects caused due to nanotherapy. Further, a subgroup analysis was performed for primary and recurrent GBM. Due to the heterogeneous nature of the treatment arm, only qualitative analysis was performed.

### Quality Assessment

For each article, the level of evidence was evaluated based on the 2011 Oxford Centre For Evidence-Based Medicine guidelines, and risk of bias independently assessed by two reviewers (H.K. and F.A.) using the Joanna-Briggs Institute (JBI) checklists [29].

## 3. Results

**Figure 1:**
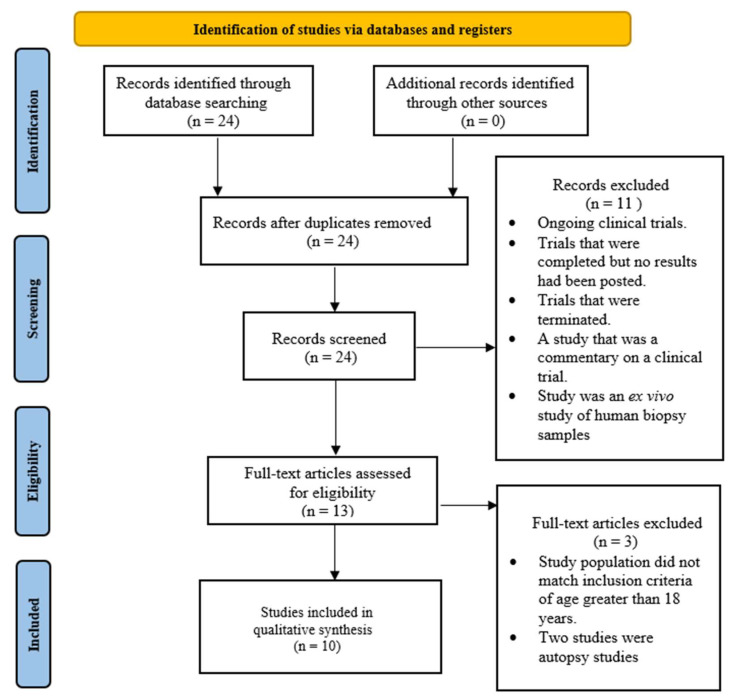
PRISMA flowchart for selection of studies.

A total of 10 studies were included for qualitative analysis (Figure 1). Of these studies, there was one non-randomized controlled trial (Level 2) and one case report (Level 4), one case series (Level 4), and seven quasi-experimental studies (Level 3). Critical appraisal of these studies revealed that two of the ten studies were of high quality according to the checklist, while the remaining eight were of medium quality, according to the JBI checklists.

### 3.1. Baseline Characteristics of Patients

**Figure 2:**
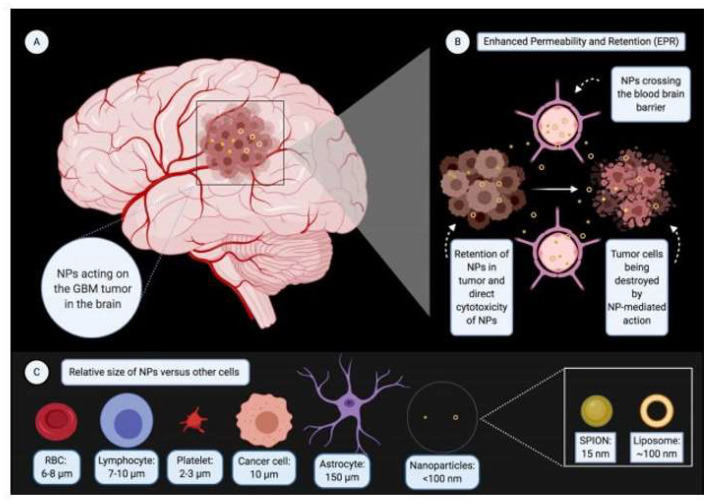
Nanotherapy for GBM. (**A**) Nanoparticles (NPs) acting on the GBM tumor in the brain. (**B**) Nanoparticles crossing the blood–brain barrier and acting on tumor cells leading to their death. (**C**) Relative size of NPs versus other cells of the body. (Figure 2 created with BioRender.com).

A total of 225 patients suffering from GBM were included in the analysis. Detailed baseline characteristics are depicted in Table 1 and Table 2. These 225 patients were further divided into two sub-populations depending on the type of GBM that was being treated, either primary or recurrent. Liposomes and superparamagnetic iron oxide nanoparticles (SPIONs) were the most employed nanoparticles [29,30,31,32,33,34,35,36,37,38]. A novel technology employing minicells synthesized from *Salmonella typhimurium* was used in a single study [39]. The size of nanoparticles ranged from 15 to 400 nm (Figure 2).

#### 3.1.1. Primary GBM

Sixty-six patients were pooled into this sub-population (Table 1) [30,33,38]. The number of males and females in this sub-population was 42 (63.64%) and 24 (36.36%), respectively. The median age of patients was 54 years. Molecular markers of glioblastoma such as methylated O6-methylguanine-DNA methyltransferase (MGMT) promoter methylation was seen in 16 out of the 33 patients evaluated (48%) and expression of immunological markers such as TNF-a, IL-1β, and IL-6 was considered in one patient [33,38]. The time of induction of therapy after the diagnosis of GBM varied from study to study and ranged from one to sixteen months (Table 1). Patients had undergone treatment for GBM before the initiation of clinical trials that included surgery, radiotherapy, chemotherapy, and immunotherapy (Table 1). The median KPS score at the beginning of the trial was 90 for patients with primary GBM.

#### 3.1.2. Recurrent GBM

One hundred and fifty-nine patients with recurrent GBM were pooled into this sub-population (Table 2) [30,31,32,34,35,36,37,39]. The number of males and females in this sub-population was 90 (59.60%) and 63 (39.62%), respectively. The median age was 52.5 years in the sub-population. Molecular markers of glioblastoma such as MGMT methylation were seen in two of the six patients evaluated (33.3%); epidermal growth factor receptor (EGFR) expression was seen in 14 patients; expression of tumor markers for drug resistance such as multidrug resistance protein-1 (MDR-1) and multiple resistance protein (MRP) was seen in 28 patients; and expression of Nestin, Glial fibrillary acidic protein (GFAP), Ki-67, and immunological markers such as CD133, CD140, and TUJ-1 were evaluated in two patients (Table 2). The time of induction of therapy after the diagnosis of GBM varied from study to study, and ranged from 24 to 54 weeks (Table 2). Patients had undergone treatment for GBM before the initiation of clinical trials that included surgery, radiotherapy, chemotherapy, and immunotherapy (Table 2). The median KPS score at the beginning of the trial was 80 for patients suffering from recurrent GBM. Overall, a large majority of patients had recurrent disease (70.67%). There was no gender bias. There was no uniformity in reporting of baseline KPS, the timing of the induction of nanotherapy, the duration of therapy, or the reporting of the molecular and immunological profiles. Primary GBM patients had higher KPS at the start of the treatment.

### 3.2. Clinical Outcomes

The pooled sample of 225 patients was analyzed further to determine the primary and secondary outcomes, based on the type of GBM. The duration of treatment and the follow-up period differed with no standard protocol followed across studies. Only two patients out of 225 patients were lost to follow-up from the pooled sample.

#### 3.2.1. Primary GBM

The primary outcome of median overall survival (mOS) was calculated to be 6.75 months for patients with primary GBM. The mean PFS period was 2.3 months, and the mean PFS-12 rate was 30.2%. Individual PFS values are listed in Table 3. The median KPS (after treatment) was 80, indicating a reduction in QoL after treatment. As per Macdonald criteria, 3.03% had a complete response, 4.54% had a partial response, and 63.64% had stable disease, after nanotherapy.

#### 3.2.2. Recurrent GBM

The primary outcome of median overall survival (mOS) was calculated to be 9.7 months in this sub-population. The mean PFS period was 3.92 months and the mean PFS- 12 rate was 8.2%. Individual values are listed in Table 4. The median KPS (after treatment) was 90, indicating an improvement in the QoL. As per Macdonald criteria, 0.63% had a complete response, 5.66% had a partial response, and 9.43% had stable disease, after nanotherapy.

### 3.3. Side Effects Profile

The side effects that were caused due to nanotherapeutic interventions are listed in Table 5. The event rate of side effects in all patients was 14.2% (*n* = 32). Life-threatening side effects in these patients included pulmonary embolism (0.44%), cerebral edema (6.67%), pneumonia (4%), mucositis (5.78%), hypophosphatemia (3.11%), and thermal stress in the brain because of nanoparticle application (3.11%). The most common side effects noted in both subgroups of primary and recurrent GBM were myelotoxicity (32.44%), vomiting or nausea (15.56%), and Palmoplantar Erythrodysesthesia (PPED) (12.89%). The number of adverse events occurring in patients with primary GBM was *n* = 18 and that in recurrent GBM patients was *n* = 22. The incidence ratio of side effects in primary GBM patients was 1.54 and that in recurrent GBM patients was 1.88. Thus, recurrent GBM patients treated with nanotherapy had a 1.22-fold higher risk of being affected by side effects.

## 4. Discussion

GBM continues to be the deadliest form of brain cancer, with a mere median survival of 15 months. GBM remains incurable and resistant to treatment despite the use of the latest technologies. The gold standard therapy for GB for at least two decades has been the Stupp regimen, which constitutes treatment with radiation, continuous daily temozolomide, followed by adjuvant daily temozolomide [2]. Adjunct therapies for GBM include the use of monoclonal antibodies, such as Bevacizumab [40]. However, overall survival and progression-free survival are not considerably better. Invasive injection, transient BBB disruption, and the use of drug delivery systems are approaches used to transport drugs to the brain [41]. Therapeutic agents can enter the brain when the blood–brain barrier is temporarily disrupted. Therefore, leveraging endogenous transport mechanisms for drug delivery across the BBB, which are less invasive, presents a more appealing entry route. Treatment failure may be attributed to a combination of elements such as acquired drug resistance, intrinsic unresponsiveness, and limited brain tumor accessibility to drugs associated with the blood–brain barrier’s (BBB) impermeability. Furthermore, glioblastoma has evolved a variety of mechanisms that suppress or impede the anti-tumor immune responses, which is probably an additional contributor in the failure [42]. Hence, there is a significant need for innovative therapeutic approaches to enhance the outcomes currently observed with conventional therapies.

A less invasive technique for delivering a targeted medication to a brain tumor is ultrasound-assisted brain delivery [43]. A combination of conventional chemotherapy medications and ultrasound-assisted brain delivery, gene therapy, NPs, and antibodies greatly improve brain uptake and therapeutic success in numerous instances [44,45,46,47]. Uncertainty was raised over the practical application of ultrasound-mediated immunotherapy when it was discovered that, in certain instances, the delivery of a particular monoclonal antibody by ultrasound enhanced brain uptake but did not increase therapeutic efficacy [48]. The rate of drug administration to precisely defined tumor tissues is increased when imaging methods are used with focused ultrasound (FUS). To achieve a greater tissue delivery of temozolomide in mice, liposome-encapsulated doxorubicin in rats, and cisplatin-conjugated gold nanoparticles in mice, MRI-guided FUS (MRgFUS) was employed [49,50,51]. Rats with experimentally developed gliomas had a higher overall survival rate and more effective local distribution of temozolomide to tumors when the blood–brain barrier (BBB) was disrupted by FUS [49]. Rats with FUS application and BCNU administration had better survival rates and a slower rate of tumor progression [52]. Low-intensity fluorescence ultrasound (LIFU) was employed to deliver a liposomal O6-(4-bromothenyl) guanine (O6BTG) derivative that inactivates MGMT in a mouse model with temozolomide-resistant glioma [53]. Sadly, there are a number of potential side effects associated with ultrasound-mediated disruption of the blood–brain barrier, such as hemorrhagic change [54], edema [55,56,57], inflammation [57,58], neuronal ischemia [59], and tissue apoptosis [58,59]. When dealing with disorders that have a longer prodromal phase, invasive techniques provide challenges due to their high maintenance costs and follow-up requirements, which can lead to patient noncompliance. They also have downsides such as inadequate medication penetration outside of the resection cavity and restriction of drug dosage by the implant’s size, association with elevated intracranial pressure, and local toxicity resulting in brain damage and infections [60]. Consequently, non-invasive delivery techniques without those disadvantages have been created.

Recent developments in nanotechnology have resulted in the emergence of nanoparticles (NPs) with the ability to pass through glioma cell membranes, effectively transport medications to the tumor location, and bypass the blood–brain barrier [61,62]. They should be smaller than 200 nanometers, or possibly even smaller based on the structure, chemical makeup, surface charge, and nanomechanical characteristics [63,64]. NPs must induce receptor-mediated transcytosis, which can be induced by certain glycoproteins and antibodies, in order to pass across the blood–brain barrier. Encasing anticancer medications into carbohydrate polymer NPs and directing them toward the tumor cells is one method of employing these NPs for the treatment of gliomas [65]. It has been demonstrated that GBM is a non-T cell-inflamed cancer characterized by an immunosuppressive microenvironment and high immune escaping ability [66]. Cell dysfunction and the dearth of glioblastoma-infiltrating T cells are likely factors in the tumors’ resistance to single-modality immunotherapy. Several studies have been carried out in an attempt to overcome this lack of response by converting immunologically “cold” tumors into “hot” tumors [67,68]. A study reports remarkable results with the use of SGT-53 combined with Anti-PD-1 immunotherapy in mouse models of glioblastoma [69]. A plasmid encoding human wild-type TP53 (wtp53) is enclosed in the unique cationic liposome known as SGT-53. A single chain antibody fragment that recognizes the transferrin receptor (TfRscFv) and specifically targets cancer cells overexpressing TfR is attached to the liposome surface [70]. It was reported that Anti-PD-1 and the studied nanomedicine SGT-53 worked well together to increase intratumoral T-cell infiltration, induce tumor cell apoptosis, and suppress tumor development. Mice with intracranial glioblastoma undergoing combined therapy showed a notable improvement in survival. Crucially, SGT-53 increased the expression of PD-L1 in vivo and in vitro. A plasmid encoding human wild-type TP53 (wtp53) is enclosed in the unique cationic liposome known as SGT-53. A single chain antibody fragment that recognizes the transferrin receptor (TfRscFv) and specifically targets cancer cells overexpressing TfR is attached to the liposome surface [69]. Transferrin is a serum iron carrier protein that interacts with the luminal transmembrane glycoprotein, transferrin receptor 1 (TfR1), thereby regulating the uptake and transport of iron across the blood–brain barrier (BBB) for neural conductivity and metabolism [71]. In normal physiological conditions, TfRs selectively bind and facilitate the entry of endogenous transferrin, while excluding many drugs and recombinant proteins [72]. GBM tumor cells highly overexpress TfRs [73], presenting an opportunity to exploit these receptors as a target for systemic nanoparticle (NP) drug delivery. This can be achieved by generating antibodies against TfRs and using transferrin as a ligand-targeting moiety [72]. There are some preclinical studies that support the clinically applicable labeling of nanomedicine. Gao et al. conducted a study where they conjugated IL-13 peptide (IL-13p) onto PEG-PCL nanoparticles (ILNPs) to specifically target the IL13Rα2 receptor, which is exclusively expressed by all cancerous cells [74]. The IL-13p peptide ligand was found to possess cell-penetrating characteristics, enhancing specificity and facilitating cellular uptake through receptor-mediated endocytosis. A fluorescent model drug, Coumarin-6, was loaded into PEG-PCL nanoparticles, and ILNPs were intravenously administered to U87 xenograft-bearing BALB/c nude mice. The nanoparticle tumor bio-distribution revealed that ILNP fluorescence was 2.96-fold higher than that of the PEG-PCL nanoparticle treatment group, demonstrating the enhanced delivery of ILNPs through receptor targeting. Friden et al. successfully intravenously delivered methotrexate (MTX) across the Sprague–Dawley rat blood–brain barrier (BBB) using the anti-transferrin receptor monoclonal antibody (OX-26). This approach allowed for the selective targeting of cells expressing transferrin receptors (TfR) [75]. The authors noted a higher uptake of the labeled antibody with the OX-26-MTX conjugate 24 h post-injection compared to the antibody alone. Kang et al. conducted a study where they conjugated the CRTIGPSVC (CRT) peptide, mimicking iron binding to a complex of transferrin (Tf)/TfR, to poly(ethylene glycol)-poly(L-lactic-co-glycolic acid) nanoparticles (CRT-NPs). In BALB/c nude mice bearing intracranial C6 glioma, they administered Coumarin-6-labeled nanoparticles, CRT-NPs, and Tf-NPs via tail vein injection. CRT-NPs demonstrated higher levels of penetration and accumulation at the tumor site compared to Coumarin-6-labeled nanoparticles and Tf-NPs alone (2.41-fold and 1.43-fold change, respectively) [76]. This highlights the potential of the CRT peptide as a targeting ligand for enhanced drug delivery in glioblastoma.

Our systematic review is the first comprehensive review of the various types of modern technologies in use for the treatment of GBM, spanning over 2 decades. Nanoparticles have emerged as a possible replacement for traditional modalities for diagnosing and treating several types of cancer [77,78]. The benefit over traditional medicine appears to be the targeted approach towards tumor cells, better penetration into tumor tissues, and therapies designed specifically to cross the BBB, such as micelles and liposomes [23,25]. In addition to nanotherapeutic approaches, we also compared the outcomes to prior data from therapy using Bevacizumab, a monoclonal antibody drug that inhibits the VEGF pathway and has been widely used for adjunct GBM therapy [40,79].

While there are numerous types of NPs being used for GBM therapy, diagnostics, and theranostics in pre-clinical studies, only a few have been implemented in clinical use (Table 6).

In our systematic review, we observed 225 patients who underwent adjuvant nanotherapy. The median overall survival (mOS) after pooled analysis was calculated to be 8.2 months (as opposed to the mOS of 12.5 months with Bevacizumab monotherapy). Median overall KPS before therapy was 80, and after therapy was 90 in patients. This showed an overall improvement in the quality of life of patients. Patients in the recurrent group had relatively better KPS after the treatment. The mean PFS-12 rate was 19.2%, and the mean PFS was 3.11 months for patients undergoing adjunct nanotherapy (compared to a mean PFS of 10.7 months (primary GBM) and, in the recurrent GBM group, a mOS of 9.3 months with Bevacizumab therapy).

It is surprising that, overall, clinical outcomes for recurrent GBM are better than those for primary disease. This difference can be attributed to the heterogeneity of the disease, such as the location or size of the tumor, aggressiveness of the tumor, and molecular profile of the tumor. However, the better outcomes associated with recurrent GBM, along with preserved or improved KPS, certainly show the availability of an option that could offer prolonged survival with a reasonable quality of life, particularly when other options are scarce.

When analyzed for adverse events, 35 adverse events owing to nanotherapy were observed in 225 patients (event rate = 15.6%), of which six were serious side effects, including pulmonary embolism, cerebral edema, pneumonia, mucositis, hypophosphatemia, and thermal stress in the brain due to nanoparticle application. The number of adverse events seen in Bevacizumab-treated patients was 47, and the incidence rate of life-threatening serious adverse events was 10.6% (as compared to 5.8% in nanotherapy). The statistical analysis thus points to better efficacy with Bevacizumab compared to NP for GBM; however, it does come at a greater cost in the form of adverse events. There is a need for improved comparative investigations to determine the better modality of the two. Such investigations may pave the way toward an optimal treatment regimen for GBM.

In subgroup analysis, the efficacy of nanotherapy was shown to be better in patients with recurrent GBM (vs. primary GBM) where mOS and mean PFS were 9.7 months (vs. 6.75 months) and 3.92 months (vs. 2.3 months), respectively (Table 7).

The mean PFS-12 rate of primary GBM patients was, however, better (30.2% in primary vs. 8.2% in recurrent), and this discrepancy may have occurred because this parameter was evaluated in only one primary GBM study, whereas PFS-12 rates were recorded in multiple recurrent GBM studies, causing heterogeneity.

Another important point to be noted is that MGMT methylation was evaluated in only 17.3% of the patients, of which only 46.5% were positive for methylation of the promoter. In one of the studies, the OS-1 rate of patients with primary GBM in the methylated group was 68.8% as compared to 41.2% in the unmethylated group [33]. In another study, the mOS for patients with recurrent GBM in the methylated group was 19.2 months, and it was 7.1 months for the unmethylated groups [32]. These data could not be pooled because of the difference in the parameters (rate of survival and mOS), but they indicate a better survival in patients with a positive methylated MGMT promoter status. However, a higher proportion of patients must be evaluated for MGMT methylation as it is an essential prognostic biomarker that indicates a better response to treatment for GBM [88,89,90,91,92,93].

### Limitations

The gaps in the information available and the conformity of trial criteria are too large to allow a robust comparative analysis to be carried out. The inclusion criteria employed during study selection and after critical appraisal ensured studies with low risk of evidence were not included, hence reducing the number of studies. The data gap is due to the non-uniformity of the methodology, reporting of the trials, and the type of nanoparticles being used. To assess the true efficacy of nanotechnology, multicenter trials with uniform methodology need to be employed in patients with GBM.

## 5. Conclusions

The analysis of 225 GBM patients from 10 clinical trials treated with nanotherapy showed a median overall survival of 8.2 months, a mean PFS-12 rate of 19.2%, and a mean PFS period of 3.11 months. There is evidence of improvement in the quality of life owing to the increase in KPS scores before and after treatment. Nanotherapy modestly increases survival with fewer serious side effects; however, there is an inferiority in terms of overall survival compared to other alternatives such as Bevacizumab.

Currently, it is very premature to emphasize that nanomedicine-based therapeutics have significantly impacted clinical outcomes. There is a need for improvement in investigation protocols to overcome the heterogeneity in terms of the type of nanoparticles employed, time of induction of therapy, review protocols, and the follow-up period of the patients in the trials.

## Figures and Tables

**Table 1:** Baseline characteristics of patients with GBM treated with nanotherapy in sub-populations of patients with primary GBM. KPS before treatment, type of nanoparticles, mode of treatment, and simultaneous treatment therapy for each study are mentioned.

**No. of Patients**	63	1	2	Total = 66
**Male**	40	0	2	Total = 42
**Female**	23	1	0	Total = 24
**Median Age (Range) (In years)**	54 (30–73)	31	55 (35–73)	Final Median Age = 54
**Molecular Markers**	MGMT methylation	TNF-α, IL-1, 1β, IFN-β, IL-6	N/A	N/A
**Induction of Therapy from Diagnosis**	Within 4 weeks	16 months	N/A	N/A
**Prior Treatment**	Surgery	Surgery, radiotherapy, chemotherapy, and immunotherapy	Surgery, radiation, chemotherapy	N/A
**KPS before Treatment**	90	50	70	Median = 90
**Type of NP**	Pegylated Liposome	Cationic liposome	SPIONs	N/A
**Drug Encapsulation/Mode of Treatment**	Doxorubicin encapsulation (Caelyx TM, PEG-Dox)	In vivo transduction with human interferon β-gene (gene delivery)	Intracranial thermotherapy by amino silane-coated iron oxide NPs	N/A
**Simultaneous Standard Therapy**	Prolonged temozolomide chemotherapy and radiotherapy	Surgery	External beam radiotherapy	N/A
**References**	[33]	[38]	[30]	N/A

**Table 2:** Baseline characteristics of patients with GBM treated with nanotherapy in sub-populations of patients suffering from recurrent GBM. KPS before treatment, type of nanoparticles, mode of treatment, and simultaneous treatment therapy for each study are mentioned.

**No. of Patients**	28	31	7	12	59	6	14	2	Total = 159
**Male**	20	18	5	7	32	N/A	7	1	Total = 90
**Female**	8	13	2	5	27	N/A	7	1	Total = 63
**Median Age (Range) (in years)**	53 (27–68)	50 (21–70)	43 (26–65)	55 (35–73)	55.7	60 (42–75)	2	38.5 (30–47)	Final Median Age = 52.5
**Molecular Markers**	Multidrug resistance protein 1 (MDR-1) and Multiple resistance protein (MRP)	N/A	N/A	N/A	N/A	MGMT methylation	EGFR expression	Nestin; GFAP; Ki-67; CD133; CD140; TUJ-1	N/A
**Induction of Therapy from Diagnosis**	N/A	N/A	N/A	N/A	N/A	N/A	N/A	54 weeks for patient 1; 24 weeks for patient 2	N/A
**Prior Treatment**	Surgery; radiation; chemotherapy	Surgery; radiation; chemotherapy	Surgery; radiation; chemotherapy	Surgery; radiation; chemotherapy	Surgery; radiation; chemotherapy	Not reported	Chemotherapy	Surgery; radiation; chemotherapy	N/A
**KPS before Treatment**	80	80	80	70	90	N/A	N/A	100	Median = 80
**Type of NP**	Pegylated Liposome	Pegylated Liposome	Liposome	SPIONs	SPIONs	SPIONs	Minicell (VED Vox) (EnGeneIC)	Liposome (DepCyt)	N/A
**Drug Encapsulation/Mode of Treatment**	Doxorubicin encapsulation (Caelyx TM, PEG-Dox)	Doxorubicin encapsulation (Caelyx TM, PEG-Dox)	Doxorubicin encapsulation (Caelyx TM)	Intracranial thermotherapy by aminosilane-coated iron oxide NPs	Intratumoral thermotherapy by aminosilane-coated iron oxide NPs	Intracavitary thermotherapy by aminosilane-coated iron oxide NPs	Doxorubicin encapsulation; EGFR targeting via Vectibix	Cytarabine encapsulation-Intraventicular administration	N/A
**Simultaneous Standard Therapy**	Alone or in combination with tamoxifen	Alone or in combination with temozolomide	None	External beam radiotherapy	External beam radiotherapy	Concurrent, fractionated radiotherapy	None	None	N/A
**References**	[35]	[34]	[36]	[30]	[31]	[32]	[39]	[37]	

N/A is for not available.

**Table 3:** Clinical outcomes of patients with GBM treated with nanotherapy in sub-populations of patients with primary/newly diagnosed GBM. KPS after treatment, PFS rate, and Macdonald criteria for each study are quoted.

Type of NP	Pegylated Liposome	Cationic Liposome	SPIONs	
Drug Encapsulation/Mode of Treatment	Doxorubicin encapsulation (Caely TM, x PEG-Dox)	In vivo transduction with human interferon β-gene (gene delivery)	Intracranial thermotherapy by amino silane-coated iron oxide NPs	
Simultaneous standard therapy	Prolonged temozolomide chemotherapy and radiotherapy	Surgery	External beam radiotherapy	
Duration of Treatment	8 weeks	28 days = 4 weeks	N/A	
Follow-up period	20 weeks	3 years until death	3-monthly	
Patients lost to follow-up	1 (included in statistical analysis)	N/A	N/A	Total = 1
Mos in months	mOS = 17.6; [OS-24 = 35.3%]	22 weeks (5.1 months approx.)	OS-1 = 3 months (Patient 1); 8.4 months (Patient 2)	Median mOS = 6.75 months
PFS rate	PFS = 12 = 30.2%	10 weeks = 2.3 months	TTP 1 = 4.5 TTP 2§ = 5.9 months	Mean PFS-12 = 30.2%; Mean PFS = 2.3 months
KPS after treatment	85	70	N/A	Median = 80
Macdonald criteria	2 CR; 3 PR; 41 SD	1 SD	N/A	Total = 2 CR, 3 PR, 42 SD
Type of Trial	Phase-I/II trial; non-randomized; non-controlled; multi-center trial; non-randomized; non-controlled; multi-center	Non-randomized; non-controlled; single-arm	Non-randomized; non-controlled; single-arm	
Level of Evidence	4	4	4	
Reference	[33]	[38]	[30]	

**Table 4:** Clinical outcomes of patients with GBM treated with nanotherapy in sub-populations of patients with recurrent GB. KPS after treatment, PFS rate, and Macdonald criteria for each study are quoted.

Type of NP	Pegylated Liposome	Pegylated Liposome	Liposome	SPIONs	SPIONs	SPIONS	Minicell (VEDVDox) (EnGeneIC)	Liposome (DepoCyt)	
Drug encapsulation/Mode of Treatment	Doxorubicin encapsulation (Caely TM, x PEG-Dox)	Doxorubicin encapsulation (Caely TM, x PEG-Dox)	Doxorubicin encapsulation (Caelyx TM)	Intracranial thermotherapy by aminosaline-coated iron oxide NPs	Intratumoral thermotherapy by aminosaline-coated iron oxide NPs	Intracavitary thermotherapy by aminosaline-coated iron oxide NPs	Doxorubicin encapsulation; EGFR targeting via Vectibix	Cytarabine encapsulation—Intraventricular administration	
Simultaneous standard therapy	Alone or in combination with tamoxifen	Alone or in combination with temozolomide	None	External beam radiotherapy	External beam radiotherapy	Concurrent, fractionated radiotherapy	None	None	
Duration of Treatment	8 weeks	N/A (duration of study—5 years)	7 weeks (median calculated)	N/A	N/A	N/A	8 weeks/until disease progression	6 months (24 weeks)	
Follow-up period	3 years	N/A (Until death)	20 months	3-monthly	3-month intervals	3-monthly basis (mean = 11.8 ± 9.3 months)	until death	until death	
Patients lost to follow-up	0	0	0	N/A	1	N/A	N/A	N/A	Total = 1
mOS in months	26 weeks (6 months approx.)	7 months	37 weeks (8.5 months approx.)	OS-1 = 14.5 months; OS-2 = 7.6 months	OS-1 = 23.2 months; OS-2 = 13.4 months	8.15 months in general; mOS at first recurrence = 23.9 months; mOS at second recurrence = 7.1 months	9.7 (2.1–23.6) months	18 months	Median mOS = 9.7 months
PFS rate	PFS-6 = 15; PFS-12 = 7.5%	PFS-6 = 23; PFS-12 = 6%	PFS-12 = 15%	TTP-1 = 4.5; TTP-2 = 5.9 months	TTP-1 = 8 months	Median PFS = 6.25 months	1.6 months (0.7–11.3); PFS-6 = 2 months	N/A	Mean PFS-12 = 8.2%; Mean PFS = 3.92 months
KPS after treatment	N/A	N/A	85	N/A	N/A	N/A	N/A	70	Median = 90
Macdonald criteria	1 CR; 1 PR; 9 SD	2 PR	5 PD; 2 SD	N/A	N/A	N/A	4 SD	PR	Total = 1 CR, 9 PR, 15 SD
Type of Trial	Phase II trial; non-randomized; non-controlled; multi-arm	Non-randomized; non-controlled; multi-arm	Non-randomized; non-controlled; single-arm	Non-randomized; non-controlled; single-arm	Phase II; Non-randomized; non-controlled; single-arm	Non-randomized; non-controlled; single-arm	Phase I; Non-randomized; non-controlled; single-arm	case report; non-randomized; non-controlled; single-arm	
Level of Evidence	4	4	4	4	4	4	4	4	
Reference	[35]	[34]	[36]	[30]	[31]	[32]	[39]	[37]	

N/A is for not available.

**Table 5:** Severe adverse events and side effects profile (encompassing gut, bone marrow, and circulatory system) of primary and secondary GBM patients treated with nanotherapy.

Major Type	Severe Adverse Events/Side Effects	Primary GBM (*n* = 66)	Secondary GBM (*n* = 159)	Pooled (*n* = 225)
Gastrointestinal	Vomiting/nausea	4 (6.06%)	31 (19.5%)	35 (15.56%)
	Stomatitis	2 (3.03%)	0	2 (0.89%)
	Gastritis	2 (3.03%)	0	2 (0.89%)
	Diarrhea	3 (4.54%)	0	3 (1.33%)
Myelotoxicity	Leukopenia, lymphopenia, thrombocytopenia, neutropenia, anemia	55 (83.33%)	18 (11.32%)	73 (32.44%)
Thromboembolic	Deep vein thrombosis	2 (3.03%)	1 (0.63%)	3 (1.33%)
	Pulmonary embolism	1 (1.52%)	0	1 (0.44%)

**Table 6:** Nanotechnologies employed in clinical studies for glioblastoma, with type of intervention used are stated.

Nanotechnology	Type of Intervention	Reference
NU-0129: Spherical Nucleic Acid (SNA) platform consisting of nucleic acids arranged on the surface of a small spherical gold nanoparticle that targets cancer cells, via the BBB, to inhibit the activity of the Bcl2L12 gene to induce apoptosis	Therapeutic—siRNA delivery	[80]
SGT-53: complex of cationic liposome encapsulating a normal human wild-type p53 DNA sequence in a plasmid backbone for delivery to tumor cells via the BBB.	Therapeutic—drug (Temozolomide) and gene Delivery	[81]
2B3- 101: Glutathione pegylated liposomal doxorubicin hydrochloride	Therapeutic—drug delivery	[82]
BrUOG 329 (Onivyde): Nanoliposomal Irinotecan with enhanced ability to cross the BBB	Therapeutic—drug delivery	[83]
NanoBB- 1-Dox: nanoparticle-based formulation of doxorubicin, which enables passage of the drug across the BBB and delivery to the tumor inside the brain	Therapeutic—drug delivery	[84]
NanoTherm^®^: superparamagnetic iron oxide nanoparticles (SPIONS)	Therapeutic—Hyperthermia	[30,31,32,85]
EnGeneIC delivery vehicle (EDV): Novel nanoparticle (minicell) made from *Salmonella typhi* that encapsulates doxorubicin and targets Epithelial growth factor receptor (EGFR) by Vectibix	Therapeutic—drug delivery	[39]
Pegylated liposomal doxorubicin (Caelyx™, PEG-Dox)	Therapeutic—drug delivery	[33,34,35]
Myocet^®^: a non-pegylated liposomal doxorubicin	Therapeutic—drug delivery	[86]
Doxorubicin-loaded Anti-EGFR-immunoliposomes (C225-ILs- dox) in High-grade Gliomas (GBM-LIPO)	Therapeutic—drug delivery	[87]

**Table 7:** Comparison of efficacy of nanotherapy vs Bevacizumab. The efficacy of nanotherapy is shown to be better in patients with recurrent GBM.

Type of Therapy	Nanotherapy			Bevacizumab		
Type of GB	Primary	Recurrent	Overall	Primary	Recurrent	Overall
No. of Patients	66	159	225	637	548	1185
mOS	6.75 months	9.7 months	8.2 months	15.7 months	9.3 months	12.5 months
Mean PFS	2.3 months	3.92 months	3.11 months	10.7 months	-	-
Macdonald criteria	CR = 3.03%, PR = 4.54%, SD = 63.64%	CR = 0.63%, PR = 5.66%, SD = 9.43%	CR = 1.33%, PR = 5.33%, SD = 25.33%	-	CR = 6.02%, PR = 49.09%, SD = 29.02%	-

mOS = median Overall Survival, PFS = Progression-Free Survival, CR = Complete Remission, PR = Partial Remission, SD = Standard Deviation.

## Data Availability

Not applicable.
